# An Unguided, Computerized Cognitive Behavioral Therapy Intervention (TreadWill) in a Lower Middle-Income Country: Pragmatic Randomized Controlled Trial

**DOI:** 10.2196/41005

**Published:** 2023-04-26

**Authors:** Arka Ghosh, Rithwik J Cherian, Surbhit Wagle, Parth Sharma, Karthikeyan R Kannan, Alok Bajpai, Nitin Gupta

**Affiliations:** 1 Department of Biological Sciences and Bioengineering Indian Institute of Technology Kanpur Kanpur India; 2 Department of Cognitive Science Indian Institute of Technology Kanpur Kanpur India; 3 Institute of Physiological Chemistry University Medical Center Mainz Mainz Germany; 4 Department of Computer Science and Engineering Indian Institute of Technology Kanpur Kanpur India; 5 Counseling Service Indian Institute of Technology Kanpur Kanpur India; 6 Mehta Family Center for Engineering in Medicine Indian Institute of Technology Kanpur Kanpur India

**Keywords:** computerized cognitive behavioral therapy, cCBT, depression, digital intervention, mobile phone

## Abstract

**Background:**

Globally, most individuals who are susceptible to depression do not receive adequate or timely treatment. Unguided computerized cognitive behavioral therapy (cCBT) has the potential to bridge this treatment gap. However, the real-world effectiveness of unguided cCBT interventions, particularly in low- and middle-income countries (LMICs), remains inconclusive.

**Objective:**

In this study, we aimed to report the design and development of a new unguided cCBT–based multicomponent intervention, TreadWill, and its pragmatic evaluation. TreadWill was designed to be fully automated, engaging, easy to use, and accessible to LMICs.

**Methods:**

To evaluate the effectiveness of TreadWill and the engagement level, we performed a double-blind, fully remote, and randomized controlled trial with 598 participants in India and analyzed the data using a completer’s analysis.

**Results:**

The users who completed at least half of the modules in TreadWill showed significant reduction in depression-related (*P*=.04) and anxiety-related (*P*=.02) symptoms compared with the waitlist control. Compared with a plain-text version with the same therapeutic content, the full-featured version of TreadWill showed significantly higher engagement (*P=*.01).

**Conclusions:**

Our study provides a new resource and evidence for the use of unguided cCBT as a scalable intervention in LMICs.

**Trial Registration:**

ClinicalTrials.gov NCT03445598; https://clinicaltrials.gov/ct2/show/NCT03445598

## Introduction

### Background

Globally, >264 million individuals experience depressive disorders [1]. Despite the availability of evidence-based pharmacological and psychological treatment approaches, 76% to 85% of the individuals experiencing mental health disorders do not receive any treatment in low- and middle-income countries (LMICs) [2]. Barriers to accessing treatment for mental health disorders include the lack of access to treatment options, high cost, the fear of social stigma, and an inclination to self-manage the problem [3-5]. In India, there is a treatment gap of 85.2% for major depressive disorders [6].

One approach to bridging this treatment gap is to deliver computerized psychotherapy. The first therapeutic chatbot, ELIZA, was developed in 1966 [7]; it was a rudimentary program based on text rephrasing rather than evidence-based methods. The first computer-assisted cognitive behavioral therapy (CBT)–based program for depression was delivered in 1982 [8]. However, in the past 2 decades, the advent of stable internet connection and the pervasiveness of smartphones and computers have made it feasible to deploy technological interventions at scale.

Computerized CBT (cCBT) has gained traction as a viable treatment modality, with >200 trials conducted to date [9]. cCBT for depressive disorders, both guided and unguided, has been evaluated in several clinical trials worldwide. In both guided and unguided cCBT interventions, the intervention is provided by a software; in guided interventions, a guide or a coach is additionally involved who provides encouragement, technical assistance, and explanations of the intervention, whereas in a strictly unguided intervention, the user should not have any interaction with a human guide. Recent studies and meta-analyses have indicated that for depressive symptoms, guided cCBT interventions are more beneficial than unguided cCBT interventions [10-14]. Carlbring et al [15] showed equivalent effects between guided cCBT interventions and face-to-face CBT. Including guided cCBT intervention with treatment as usual does not add any extra benefits [16]. Moreover, although guided cCBT intervention can be a feasible option in high-income countries [17], it is not feasible in LMICs because of the acute shortage of mental health professionals who can act as qualified guides [18].

Unguided cCBT interventions have the potential to bridge the treatment gap in LMICs. The evidence for unguided cCBT interventions is mixed, with some meta-analyses showing that they are effective with a small or medium effect size [14,19,20] and some showing that they are not effective [21-23]. The effectiveness of the unguided interventions is reduced by the high dropout rates. Note that the unguided studies included in the meta-analyses often involved initial contact with humans for diagnostic interviews [24-29], weekly telephone contact support [30,31], or treatment as usual [16,32]. Even minimal human contact can increase adherence to the interventions compared with a study without any such contact [33,34]. Indeed, Fleming et al [35] found that adherence rates observed in trial settings failed to translate into the real world. Recent meta-analyses have reported a positive correlation between treatment adherence and treatment effects [14,19]. In addition, a recent meta-analysis found that existing guided or unguided cCBT interventions had low acceptability among patients, which was even less than that of waitlist [10].

Studies on cCBT interventions have been conducted predominantly in high-income countries [36]; however, systematic reviews on depression and mental health disorders in LMICs have been done by Martínez et al [37] and Fu et al [38], respectively. A recent meta-analysis reported that 92% of the studies on diagnosed depression had been conducted in Western Europe, North America, and Australia [39]. The interventions have been developed, evaluated, and made available for free only in these high-income regions. There is a need for unguided interventions that are more effective, have higher adherence, and are available free of cost for wide accessibility in LMICs.

### Objective

In this study, we developed and evaluated such a cCBT-based multicomponent intervention, TreadWill. We included several features in TreadWill that could increase adherence to and improve the effectiveness of a completely unguided intervention. We also developed an active control version of TreadWill that presented the same therapeutic content without these features. We designed a fully remote 3-armed randomized controlled trial (RCT)—an experimental version of TreadWill, a plain-text version of TreadWill with the same therapeutic CBT content (active control), and a waitlist control. We hypothesized that the participants in the experimental group would show significantly greater improvement in depressive and anxiety symptom severity. We also hypothesized that the participants in the experimental group would show significantly more engagement in terms of modules completed and absolute time used compared with the active control group participants.

## Methods

### Study Design

We designed a fully remote RCT to test the effectiveness of the experimental version of TreadWill compared with an active control version and a waitlist control version. We planned to recruit 600 participants with a 1:1:1 distribution across the 3 groups. We implemented simple randomization using an automated randomization function (developed in Python; version 3.4.3; Python Software Foundation). This trial was registered at ClinicalTrials.gov before commencement (NCT03445598).

### Participant Recruitment and Screening

We recruited the participants using both offline and web-based publicity. We displayed flyers in residential hostels, research buildings, and lecture halls at the Indian Institute of Technology, Kanpur. A press release helped with coverage in newspapers and social media.

The publicity material included a website link to join this study. The link opened a web page that provided information regarding the study and accepted the email ID of the interested participants. Over the next 3 steps, the web page collected the demographic data, baseline Patient Health Questionnaire-9 (PHQ-9) score [40], and informed consent from the potential participants. The entire participant recruitment process was automated (including self-reports and self-administered questionnaires) to eliminate human contact and maintain scalability. To be eligible to participate in the study, an individual must be an Indian resident aged between 16 and 35 years. They must be fluent in English and have had access to an internet-enabled computer or tablet device. They must have had scored between 5 and 19 (both inclusive) in the PHQ-9 with a score of 0 on the ninth question. We decided to include participants with mild symptoms of depression (a score of 5-9 in the PHQ-9) and exclude those with severe symptoms (a score >19) because our program was targeted not at the clinical population but at a wider population with susceptibility for depression. We excluded individuals who were unemployed, had a diagnosis of bipolar disorder or psychosis, or reported that they just wanted to check out the site and did not plan to complete it (the last condition was added after trial commencement to exclude casual visitors to the website). Because of the pragmatic nature of our study, we included participants regardless of whether they were receiving treatment for depression. Once a potential participant met the inclusion and exclusion criteria and provided informed consent (for individuals aged between 16 and 17 years, informed consent was also required from a parent or guardian), the individual was scheduled to be recruited in the study. After 18 hours, the individual was randomized to 1 of the 3 groups and received a unique link via email. The delay of 18 hours was included to prevent individuals from signing up using disposable temporary email IDs. They were counted as participants in the study only after clicking on the unique link and were led to a sign-up page (for participants assigned to the experimental or active control groups). The participants assigned to the waitlist control group were led to a page to collect their baseline Generalized Anxiety Disorder-7 (GAD-7) scores [41]; GAD-7 scores of the experimental and active control groups were taken just before the start of the first module in the intervention. The participants did not receive any monetary compensation.

### Ethics Approval

The Institutional Ethics Committee of the Indian Institute of Technology Kanpur provided ethical clearance to conduct this study (IITK/IEC/2017-18 II/1).

### Safety Check

At any stage in the intervention, if we detected severe depressive symptoms or suicidal ideation, we blocked access to TreadWill. Severe depressive symptoms were determined as a total score of >19 on the PHQ-9. Suicidal ideation was detected by a score of >0 on the ninth question of the PHQ-9 and a total score of >4 on the Suicidal Intent Questionnaire [42]. In such cases, email and SMS text messaging alerts were sent to the participants (and their *buddy*, if they had one in the program), requesting them to seek professional help. For participants aged between 16 and 17 years, an email notification was sent to the parent or guardian as well. The participants had not been informed of this exclusion criterion; therefore, they did not intentionally suppress their scores for the sake of continuing the intervention.

### Automated Notifications

Participant contact was minimal and automated. Participants who initiated the recruitment process but did not complete it were sent automated email reminders encouraging them to complete the process. Participants also received periodic automated email and SMS text messaging reminders nudging them to use TreadWill (Table S1 in Multimedia Appendix 1 provides details). The program asked the participants about their preferred time to log in; using this information, email and SMS text messaging alerts were sent 10 minutes in advance to remind the participants. The research team did not initiate any direct contact with the participants. Technical support via email was provided in case the participants sent an email requesting for it.

### Active Control Version

The active control version presented the same CBT content as the experimental version in the same 6 modules, but used plain text instead of slides, videos, and conversations. Each module had *Introduction*, *Learn*, and *Discuss* sections, but the *Practice* section was excluded. The content was not tailored according to the participant. The active control version included the CBT forms, but excluded games, such as *SupportGroup*, *PeerGroup*, and the option to involve a buddy. The participants received only essential email notifications (Table S1 in Multimedia Appendix 1 presents the details of notifications). The active control version was introduced to test whether the additional interactive elements introduced in the experimental version increased user engagement.

### Development of the Intervention

We used the Django framework (Django Software Foundation) for developing the TreadWill website. We used Google Slides (Google LLC) to embed the slides and YouTube (Google LLC) to embed the videos on the website. We used images with a Creative Commons license for use in slides and videos. We used images from the internet for the *Identify the friendly face* game [43]. The content and the website underwent multiple rounds of checking by the development team and other volunteers to fix errors before launching the trial.

### Assessments

We used the PHQ-9 [40] and GAD-7 [41] questionnaires to measure depressive and anxiety symptom severity, respectively.

For the experimental and active control group participants, the PHQ-9 and GAD-7 were administered before the beginning of each module, after completing all the modules, and at the 90-day follow-up time point. The first PHQ-9 (administered before randomization, as it was an inclusion-exclusion criterion) and the first GAD-7 (administered after randomization but before the first module of the intervention) served as the baseline scores. For the waitlist control group, the PHQ-9 and GAD-7 were administered at baseline and after a 42-day interval (this interval was chosen to be at par with the expected intervention duration of approximately 6 weeks for completing the 6 modules in the experimental group). After submitting the 42-day assessments, the waitlisted participants were also given access to the intervention.

### Blinding

All the participants followed the same recruitment procedure. Consequently, the participants were unaware of which version of TreadWill they were assigned to (they did not even know that 2 different versions existed). Therefore, we expected placebo effects in the 2 groups to be similar. In addition, the PHQ-9 and GAD-7 data were self-reported on the website; therefore, there was no scope for evaluator bias.

### Data Security and Privacy

All the participants agreed to allow their data to be used for research purposes and to be reported in a deidentified format. All participant data were transferred over Secure Sockets Layer.

The only personal identifiers provided by the participants were their email IDs and phone numbers. Before analyzing the data, all the participants’ email IDs and phone numbers were removed from the data set.

### Primary, Secondary, and Exploratory End Points

We performed a completer’s analysis (*Discussion* section). The primary end point was the final PHQ-9 score in participants who completed at least half of the intervention (3 out of 6 modules). The primary end point was decided after the trial completion but before any data analysis. We decided the cutoff point at 3 modules to ensure that all the participants were exposed to the cognitive aspect of CBT, which we introduced in the third module. We also analyzed the data of participants who had completed all the modules. A similar analysis approach based on module completion in web-based studies has been used by Christensen et al [44], Keefe et al [45], and Rollman et al [46] (the *Discussion* section elaborates on the rationale for using this analysis approach).

TreadWill was primarily designed to help individuals with depressive symptoms. Therefore, PHQ-9 was our primary outcome measure. However, as anxiety and depression are highly comorbid, we wanted to check whether the techniques presented in TreadWill also helped in the reduction of anxiety symptoms. Thus, for the experimental and the active control groups, the secondary end point was the GAD-7 score in participants who completed at least half of the intervention (3 out of 6 modules). Other secondary end points included PHQ-9 and GAD-7 scores at the 90-day follow-up.

The intermediate PHQ-9 and GAD-7 scores (after every module) and 2 surveys conducted after the module 3 and the module 6 were used as exploratory end points.

### Statistical Analyses

Owing to the high dropout rate, we did not assume the PHQ-9 and GAD-7 scores to be normally distributed; therefore, we used nonparametric statistical tests for analyzing the effectiveness of the intervention. We used the Kruskal-Wallis test for comparing the reduction in depression or anxiety symptom severity from baseline to the primary end point among the 3 groups. All the tests were 2 tailed unless otherwise mentioned. For post hoc analysis between the groups, we used the Mann-Whitney *U* test. The tests were conducted using MATLAB (MathWorks) and Python (Python Software Foundation).

Because this was the first trial of TreadWill, we did not have a prior estimate of the dropout rate and could not perform power calculations. We chose the sample size of 600 participants based on the previous studies of similar nature [16,47].

## Results

### Approach Taken for Developing the Intervention

We aimed to develop and evaluate a fully automated intervention, TreadWill, that would be engaging and effective without any expert guidance or contact. We reviewed the existing cCBT interventions before starting the development process and considered factors that may be responsible for the high dropout rates. The common shortcomings that we identified included the lack of interactive content, lack of tailoring of the content to different users, lack of peer support for users, and lack of engaging games. Different interventions addressed some of these shortcomings by including the corresponding features; however, none of them included all the features. We developed TreadWill to address these shortcomings. During the development process, we used the inputs on initial prototypes from the institute counselors and psychiatrists and from 13 pilot users (not included in the eventual trial), before finalizing the content and user experience in TreadWill. We hypothesized that TreadWill would lead to a high adherence rate and a significant reduction in depressive and anxiety symptom severity. As we did not plan to charge the users, we also expected TreadWill to be more accessible, especially in LMICs, compared with paid interventions.

### Design of TreadWill

We designed the therapeutic content of TreadWill based on CBT, using the book by Beck [48] as the primary reference. TreadWill delivered the core concepts of CBT in a structured format with 6 modules (Table S2 in Multimedia Appendix 1 shows the details) in an easily understandable language. Each module consisted of 4 sections: *Introduction*, *Learn*, *Discuss*, and *Practice*. In the *Introduction* section, an automated virtual therapist explained the importance of the module through interactive text-based dialogue. The *Learn* section included psychoeducation in the form of slides and videos. Slides consisted of multiple infographics that were presented sequentially (Figure S1 in Multimedia Appendix 1). Videos consisted of animated content with a voiceover explaining the concepts that were visible on the screen. In the *Discuss* section, the participants learned to apply the psychoeducation to real-life situations through *conversations*. These *conversations* were text-based dialogues with an automated virtual patient (Figure S2 in Multimedia Appendix 1), presented in an interactive format designed to simulate human chat. Although the conversations were preprogrammed, in many instances, the participants could choose from >1 response, thus providing some control to the user in steering the dialogue. The *Practice* section included interactive quizzes on the material covered in each module.

To ensure sequential progression through the intervention, only the first module was initially accessible to the user and the later modules were locked. After the completion of all sections in a module and a 4-day gap since its unlocking (to prevent rushing through the modules), the next module was unlocked. Steps within a module were also unlocked gradually upon the completion of the preceding steps. Each participant had a maximum of 90 days to complete the 6 modules starting from their first log-in. After 90 days, they could continue to use the modules that were already unlocked until then but could not unlock new modules. We did this to restrict participants’ exposure to new therapeutic content after 90 days and provide a clear deadline, as recommended by several studies [49-51].

### Interactive Games and CBT Forms in TreadWill

TreadWill included 2 interactive games. The *Identifying thinking errors* game was aimed at training the participants in spotting thinking errors in their negative automatic thoughts. The gameplay involved the presentation of a situation, a related negative automatic thought, and a list of 10 thinking errors from which the participant had to select one or more thinking errors present in the thought. Selecting the correct option allowed the participant to move to the next level. When an incorrect option was selected, feedback was provided along with an opportunity to try again. The *Identify the friendly face* game is based on the training paradigm developed by Dandeneau and Baldwin [52] to train participants to overcome the negative attention bias and improve their self-esteem, thereby reducing the risk of depression [53,54]. The game presented 4 images in a 2×2 grid with 3 faces showing a negative emotion and 1 face showing a positive emotion. The participant was allowed 5 seconds to find the positive image and thus increase their score. If the participant responded or if 5 seconds elapsed, a new set of images was displayed. The gameplay incentivized quick attention to positive emotions. The difficulty of the game continuously adapted to the participants’ competence: incorrect responses increased the frequency of faces with obvious emotions, and correct responses increased the frequency of faces with subtle emotions.

TreadWill provided an interactive interface to fill in the forms commonly used in CBT: *Thought record worksheet, Core belief worksheet, Behavioral experiment worksheet, Problem-solving worksheet, Prepare for setback worksheet, and Schedule activity worksheet* (Table S3 in Multimedia Appendix 1). The forms allowed participants to apply CBT techniques to their situations and save the information for future reference.

### Peer and Family Support in TreadWill

Individuals looking for support on the internet might have low social support in real life [55]. In such cases, web-based peer-based support has been shown to be effective in reducing depressive symptoms [56,57]. Keeping this in mind, we designed the *SupportGroup* and *PeerGroup* features in TreadWill to provide a social space where participants could connect with other TreadWill users and potentially help each other in solving their problems. Posts in the *SupportGroup* were visible to all the TreadWill participants. The participants could upvote or downvote posts, add comments, and send *thank you* messages to each other. *PeerGroups* were smaller groups of 10 members each, designed in such a way that the posts in a *PeerGroup* were visible only to the members of that *PeerGroup*.

We provided the participants with the option to invite a family member or a friend as their *buddy* who would receive weekly updates about the participant’s activities in TreadWill. We hypothesized that the involvement of the *buddy* would motivate the participants to complete the program. We sent an email to this *buddy* if the participant failed to use TreadWill regularly and requested them to nudge the participant.

### Content Tailoring in TreadWill

Content tailoring has the potential to increase adherence to cCBT interventions, as participants are more likely to stick with a program if they find the content relatable [50,58,59]. In TreadWill, we implemented tailoring by selecting examples in the *conversations* based on the participant’s occupation (high school students, college students, or working professionals). In addition, we tailored the *conversations* based on participants’ thoughts, beliefs, and situations in the following manner. First, we asked the participants to select relatable intermediate and core beliefs from the Dysfunctional Attitude Scale [60], negative automatic thoughts from the Automatic Thoughts Questionnaire [61], and stressful situations from a curated list. Then, we made the simulated virtual patients in the subsequent *conversations* identify with similar beliefs, thoughts, and situations, and the participant’s goal was to help the simulated patient by using the CBT techniques learned in that module.

The automated email and SMS text messaging notifications received by the users were also tailored according to their preferences (*Method*s section).

### Participants

**Figure 1:**
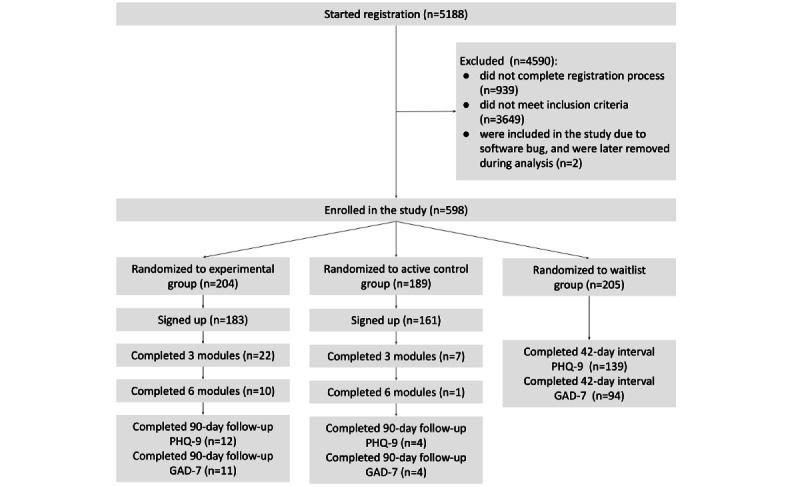
The flow of participants in the trial. In the experimental and the active control groups, the follow-up scores of only those participants who had completed at least 3 modules were analyzed. In the waitlist group, the 42-day interval scores of only those participants who had also submitted the baseline scores were analyzed. GAD-7: Generalized Anxiety Disorder-7; PHQ-9: Patient Health Questionnaire-9.

Recruitment commenced on February 14, 2018, with a planned enrollment of 600 participants. The primary completion date was March 2, 2019, after full enrollment, and the secondary completion date was May 31, 2019. Of the 5188 individuals who started the registration process for the study, 598 (11.53%) participants completed all the steps and met the study inclusion criteria (2 other participants who did not meet the inclusion criteria were initially included owing to a software bug but were excluded when we cross-checked the data during data analysis). The 598 participants were randomly assigned to the 3 study arms with equal probability (*Methods* section), resulting in 204 experimental, 189 active control, and 205 waitlist control participants (Figure 1). The participants in the 3 groups were found to be balanced in terms of age, sex, the severity of depressive symptoms, occupation, the use of other interventions, motivation for joining, and the occurrence of recent traumatic events (Table 1). The baseline PHQ-9 scores in the 3 groups were not significantly different: Kruskal-Wallis H(2)=2.04 (*P*=.36). However, a sex bias (478/598, 79.9% male) was observed because participants in our study were recruited mainly from Indian engineering colleges where the students were predominantly male [62]. In addition, in India, there is a 56% gender gap in mobile internet use [63].

**Table 1:** Baseline and demographic characteristics of the participants recruited in the study.^a^

Groups		Experimental (n=204)	Active control (n=189)	Waitlist control (n=205)	All (n=598)	Group comparison—test result					
						H(2)		Chi-square (*df*; n=598)		*P* value	
Age (years), mean (SE)		23.76 (0.30)	23.42 (0.28)	23.48 (0.29)	23.56 (0.17)	0.205		N/A^b^		.90	
**Sex, n (%)**							N/A		0.586 (2)		.75
	Male	160 (78.4)	151 (79.9)	167 (81.5)	478 (79.9)						
	Female	44 (21.6)	38 (20.1)	38 (18.5)	120 (20.1)						
**Traumatic event or death of a loved one, n (%)**							N/A		0.199 (2)		.91
	Yes	22 (10.8)	18 (9.5)	20 (9.8)	60 (10)						
	No	182 (89.2)	171 (90.5)	185 (90.2)	538 (90)						
**Joining for help, n (%)**							N/A		2.722 (2)		.26
	Yes	180 (88.2)	157 (83.1)	180 (87.8)	517 (86.5)						
	No	24 (11.8)	32 (16.9)	25 (12.2)	81 (13.5)						
**Secondary help, n (%)**							N/A		4.968 (6)		.55
	None	185 (90.7)	178 (94.2)	193 (94.1)	556 (93)						
	Counseling	5 (2.5)	4 (2.1)	4 (2)	13 (2.2)						
	Medication	12 (5.9)	4 (2.1)	6 (2.9)	22 (3.7)						
	Both	2 (1)	3 (1.6)	2 (1)	7 (1.2)						
**Occupation, n (%)**							N/A		7.620 (14)		.91
	High school Student	1 (0.5)	2 (1.1)	4 (2)	7 (1.2)						
	Between school and college	8 (3.9)	4 (2.1)	6 (2.9)	18 (3)						
	College student	113 (55.4)	103 (54.5)	120 (58.5)	336 (56.2)						
	Coaching after college	33 (16.2)	33 (17.5)	30 (14.6)	96 (16.1)						
	Working professionals	39 (19.1)	40 (21.2)	37 (18)	116 (19.4)						
	Self-employed	7 (3.4)	4 (2.1)	4 (2)	15 (2.5)						
	Freelancers	2 (1)	3 (1.6)	2 (1)	7 (1.2)						
	Volunteers	1 (0.5)	0 (0)	2 (1)	3 (0.5)						
PHQ-9^c^, mean (SE)		10.76 (0.26)	10.81 (0.25)	10.42 (0.27)	10.66 (0.15)	2.04		N/A		.36	

^a^The 3 groups were not statistically different in these characteristics, as indicated by the statistical tests reported in the last column.

^b^N/A: not applicable.

^c^PHQ-9: Patient Health Questionnaire-9.

### Effectiveness of TreadWill

In the primary analysis, we included the participants who completed at least 3 modules in the experimental group or the active control group. For this analysis, we used the last PHQ-9 scores submitted by these participants, excluding the follow-up questionnaire. Henceforth, we refer to the time of these last scores as the primary end point. In the waitlist control group, all users who submitted the questionnaires after the waiting period were included in the analysis.

**Figure 2:**
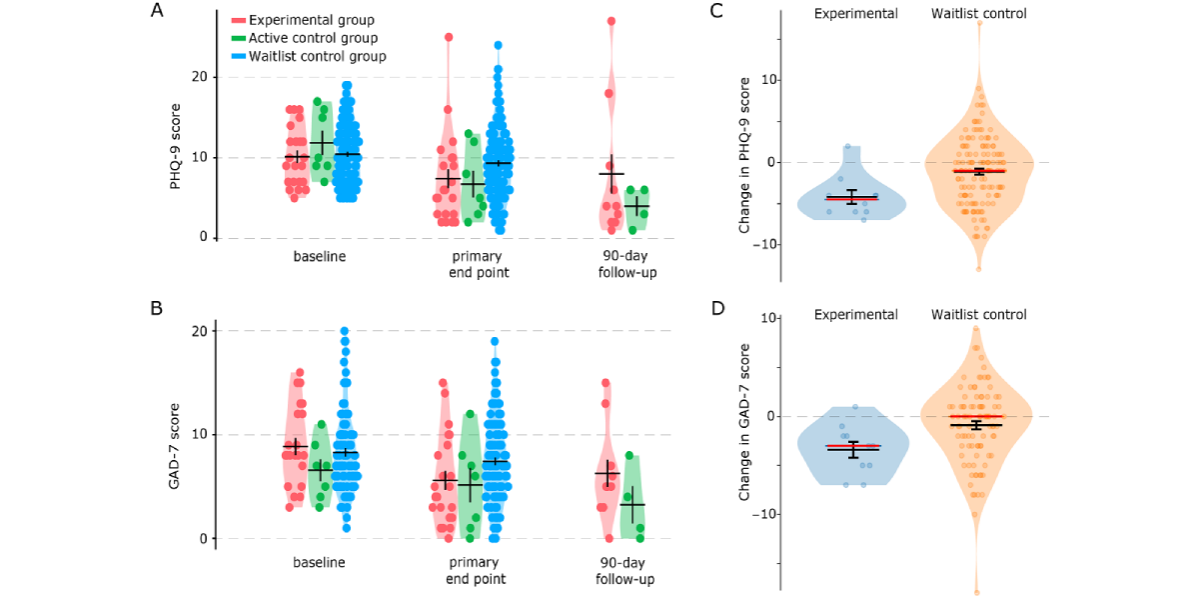
Changes in Patient Health Questionnaire-9 (PHQ-9) and Generalized Anxiety Disorder-7 (GAD-7) scores after using TreadWill. (A) and (B) Violin plots show PHQ-9 (A) or GAD-7 (B) scores at baseline, primary end point, and follow-up for the experimental group, the active control group, and the waitlist group participants. Primary end point is defined as the latest PHQ-9 or GAD-7 score submitted after completing at least 3 modules. For PHQ-9, experimental group: n=22, active control group: n=7, waitlist group: n=139; for GAD-7, experimental group: n=22, active control group: n=7, waitlist group: n=94. (C) and (D) Violin plots show the change from baseline to program completion in PHQ-9 (C) or GAD-7 (D) score for the experimental group participants who completed all 6 modules (blue violin). For waitlist group participants (orange violin), the plots show the change from the score at the baseline to the score after the 42-day waiting interval (considered as the primary end point for the waitlist group). Red horizontal lines: median; black: mean. Error bars represent SE.

We compared the reductions in the PHQ-9 scores from the baseline to the primary end point between the 3 groups (Figures 2A and 2B; Table 2). The 3 groups showed significant differences in the reductions in the PHQ-9 score (Kruskal-Wallis test H(2)=8.93; *P*=.01); a post hoc test with Bonferroni correction revealed that the experimental group showed a larger reduction than the waitlist control group (2.73 vs 1.12; Mann-Whitney *U*=1027; experimental group: n=22; waitlist control group: n=139; *P*=.04). The differences in PHQ-9 reductions between the experimental and the active control groups were not significant (*U*=96; experimental group: n=22; active control group: n=7; *P*=.34).

In secondary analysis, the 3 groups showed significant differences in the reductions in the GAD-7 score (Kruskal-Wallis test H(2)=8.02; *P*=.02); a post hoc test with Bonferroni correction showed a larger reduction in the experimental group than in the waitlist control group (3.27 vs 0.89; Mann-Whitney *U*=637.50; experimental group: n=22; waitlist control group: n=94; *P*=.02). The differences in the GAD-7 reductions between the experimental and the active control groups were not significant (*U*=52.5; experimental group: n=22; active control group: n=7; *P*=.22).

We also checked the reduction in the PHQ-9 and GAD-7 scores for the smaller set of the experimental group participants who completed all 6 modules (Figures 2C and 2D; Table 2); this analysis could not be performed for the active control group because only 1 participant from that group completed all 6 modules. This analysis also showed that the experimental group had a significantly larger reduction in PHQ-9 scores compared with the waitlist control group (4.20 vs 1.12; Mann-Whitney *U*=368.5; experimental group: n=10; waitlist control group: n=139; *P*=.01) and GAD-7 scores (3.40 vs 0.89; Mann-Whitney *U*=260.5; experimental group: n=10; waitlist control group: n=94; *P*=.02). The participants who completed all modules in the experimental and the active control groups did not differ demographically or in their baseline PHQ-9 scores from the rest of the participants (Table S4 in Multimedia Appendix 1).

The reductions observed in the PHQ-9 and GAD-7 scores in the experimental and the active control groups at the primary end point were maintained at the 90-day follow-up period (Figures 2A and 2B). Thus, both the full-featured version of TreadWill (experimental) and the plain-text version of TreadWill (active control) were effective in reducing depression- and anxiety-related symptoms in participants who completed all or at least 3 modules.

**Figure 3:**
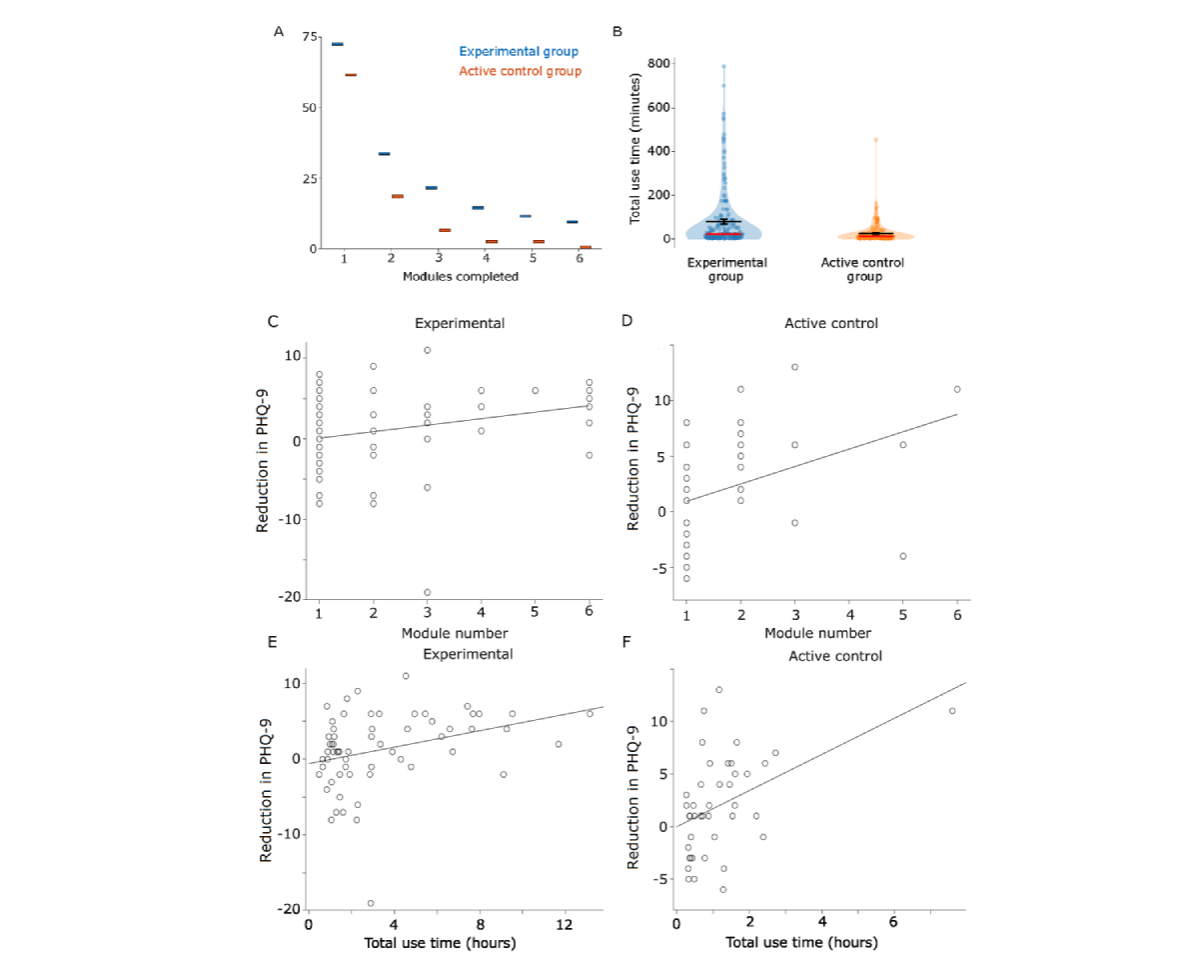
Adherence with TreadWill and the relationship between intervention use and symptom reductions. (A) The graph shows the number of participants in the experimental (blue) and the active control (red) groups who completed the indicated number of modules. (B) Violin plots show the total use times of the experimental and the active control group participants. Red horizontal lines: median, black: mean. Error bars represent SE. (C) and (D) The reduction in Patient Health Questionnaire-9 (PHQ-9) scores versus the number of modules completed by the experimental group participants (C) and the active control group participants (D). (E) and (F) The reduction in PHQ-9 scores versus the total use time in hours for the experimental group participants (E) and the active control group participants (F). In all cases, the reduction in PHQ-9 scores was calculated by subtracting the last PHQ-9 score (excluding follow-up) from the baseline score; a positive value indicates improvement. Some points in the graphs are overlapping.

We checked whether the novel features of the experimental version of TreadWill were able to increase engagement compared with the active control version. Every module was completed by more participants in the experimental version than in the active control version (Figure 3A). The odds of completing at least 3 modules were 3 times higher for a participant in the experimental group compared with a participant in the active control group (odds ratio 3.004, 95% CI 1.247-7.237; *P*=.01). The experimental group participants used TreadWill for an average of 79.8 minutes and the active control group participants for 26.1 minutes; the difference was statistically significant (Mann-Whitney *U*=10,290; experimental group, n=181; active control group, n=159; *P*<.001; Figure 3B). Thus, the full-featured version of TreadWill had higher engagement and less attrition than the plain-text version.

Furthermore, we checked whether the level of engagement with TreadWill was related to the reductions in depressive and anxiety symptoms. We found that the reduction in the PHQ-9 score was positively correlated with the number of modules completed within each group (experimental group: Spearman ρ=0.38; *P*=.003; n=61; Figure 3C; active control group: ρ=0.51; *P*<.001; n=41; Figure 3D) and with the total use time (experimental group: ρ=0.39; *P*=.002; n=61; Figure 3E; active control group: ρ=0.47; *P*=.002; n=41; Figure 3F). The reduction in the GAD-7 score was also moderately correlated with the number of modules completed (experimental group: ρ=0.27; *P*=.04; n=57; Figure S3A in Multimedia Appendix 1; active control group: ρ=0.43; *P*=.009; n=37; Figure S3B in Multimedia Appendix 1) and total use time (experimental group: ρ=0.25; *P*=.07; n=57; Figure S3C in Multimedia Appendix 1; active control group: ρ=0.35; *P*=.04; n=37; Figure S3D in Multimedia Appendix 1).

**Table 2:** Average changes (SE) in Patient Health Questionnaire-9 (PHQ-9) and Generalized Anxiety Disorder-7 (GAD-7) scores for the 3 groups from the baseline to the primary end point or to the completion of all modules.^a^

Groups			Experimental (n=204)					Active control (n=189)					Waitlist control (n=205)		
			Average change (SE)		Values, n (%)		Average change (SE)			Values, n (%)		Average change (SE)			Values, n (%)
**PHQ-9**															
	Primary end point	−2.73 (1.27)		22 (10.8)		−5.14 (2.28)			7 (3.7)		−1.12 (0.37)			139 (67.8)	
	Completion of all modules	−4.20 (0.83)		10 (4.9)		—^b^			—		—			—	
**GAD-7**															
	Primary end point	−3.27 (0.97)		22 (10.8)		−1.43 (0.92)			7 (3.7)		−0.89 (0.42)			94 (45.9)	
	Completion of all modules	−3.40 (0.82)		10 (4.9)		—			—		—			—	

^a^As only 1 participant in the active control group completed all modules, the corresponding values were not analyzed.

^b^Not available.

### Evaluating the Role of Possible Confounding Factors

Differences in time and motivation can act as confounding factors in the performance of an intervention. To test whether the observed differences between the experimental and the waitlist groups were affected by these factors, we performed additional analyses.

We had planned to take the PHQ-9 and GAD-7 scores in the waitlist group at 42 days, as the experimental group participants were also expected to take 42 days to submit the final questionnaire (6 modules at the rate of 1 module per week). However, variability in the actual timing of score submission was inevitable in a fully unguided and remote study. In our data set, we found that the actual timing of the final questionnaire was 63.7 (SD 26.8) days for the experimental group and 47.4 (SD 10.5) days for the waitlist group participants. To check if this difference in timing can explain the difference in the performance, we split the waitlist group participants into 2 subgroups depending on when they submitted the PHQ-9 questionnaires: the first subgroup included participants who had submitted before 46 days (mean 43.56, SD 1.10 days; n_WL1_=99), and the second subgroup included participants who submitted after 46 days (mean 57.05, SD 15.91 days; n_WL2_=40); by design, the mean number of days for the 2 subgroups were significantly different (Mann-Whitney *U*=3960; *P*<.001). However, the mean reductions in PHQ-9 scores for these 2 subgroups were not significantly different (*U*=1987.5; *P*=.97). This indicates that for the waitlist group, the difference in the number of days in the observed range did not affect the PHQ-9 scores significantly.

To check whether the higher reduction in the PHQ-9 scores in the experimental group than in the waitlist group can be explained by motivation, we performed the following analysis. In our study, the waitlist group participants were given the option to sign up for the experimental version of the intervention once the waitlist period was over (ie, when their formal participation in the study had ended, they were not considered as experimental group participants). It is reasonable to expect that the waitlist group participants who actually signed up for this option, despite the long gap of at least 42 days, were more motivated than the rest. We created a subgroup of these more motivated waitlist group participants and compared their performances with that of the remaining participants. These 2 subgroups did not show a significant difference in the reductions in the PHQ-9 scores (*U*=2160; motivated: n=64; unmotivated: n=75; *P*=.31).

Another potential concern is that the users who happened to improve spontaneously may be likely to complete more modules; by performing a completer’s analysis, we may be selecting for such spontaneous improvers. We performed an additional analysis to check whether this was the case in our data. On the basis of this argument, the participants who went on to complete module 3 after completing module 2 would have seen more improvement in their PHQ-9 scores at the end of module 2 compared with the participants who dropped out just after completing module 2. We compared the reductions in PHQ-9 scores (from baseline to the end of module 2) of these 2 subgroups and found no significant difference (*U*=93; dropout: n=9; continued: n=22; *P*=.81). Similarly, we compared the reductions in PHQ-9 scores (from baseline to the end of module 1) of participants who dropped out after completing module 1 and those who went on to complete the next module, and we did not find any significant difference (*U*=488; dropout: n=30; continued: n=31; *P*=.74). Thus, the idea that (spontaneous) improvement in performance encourages the participants to complete more modules is not supported by our data.

On the basis of these analyses, we conclude that the higher reduction in PHQ-9 scores observed in the experimental group can be attributed to the effect of completion of the modules, rather than differences in the timing of questionnaires or in motivation.

### Feedback on the Features of TreadWill

We programmed TreadWill to present surveys containing 15 questions using a 5-point Likert scale to quantify the participants’ feedback on various aspects of TreadWill. For example, one of the questions stated *I found the email reminders helpful*, to which the participant responded by selecting one of the following options: *strongly agree*, *somewhat agree*, *neither agree nor disagree*, *somewhat disagree*, and *strongly disagree*, which were mapped to a score of 2, 1, 0, −1, and −2, respectively (Table S5 in Multimedia Appendix 1 lists all questions). The surveys were conducted at 2 time points: after completing 3 modules and at the end of the intervention.

**Figure 4:**
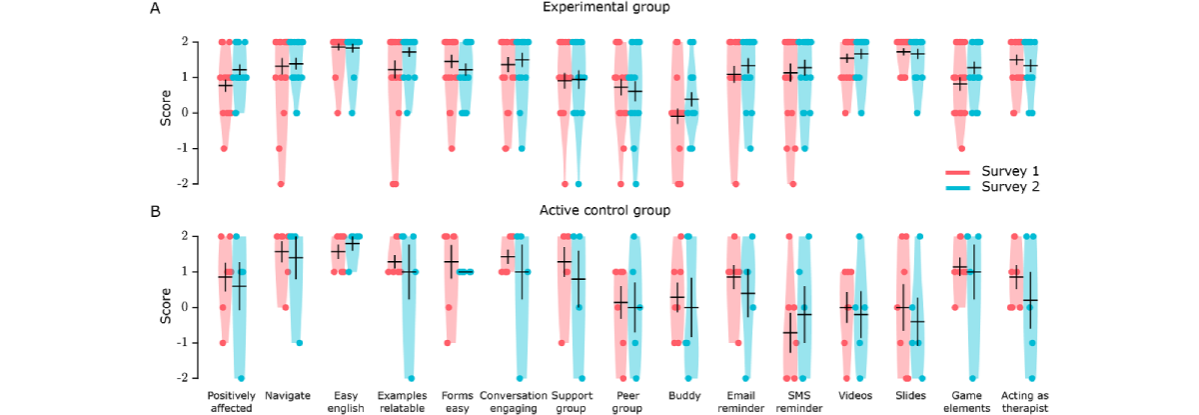
Feedback on the features of TreadWill. Violin plots show survey responses by the experimental group (A) and the active control group participants (B). Y-axis labels: 2=“strongly agree,” 1=“somewhat agree,” 0=“neither agree nor disagree,” −1=“somewhat disagree,” and −2=“strongly disagree.” Black lines indicate mean (SE).

In the experimental group, of the 22 participants who completed at least 3 modules, the first survey was submitted by 22 (100%) participants and the second survey was submitted by 18 (82%) participants. The participants reported positive feedback on most aspects of TreadWill (Figure 4A): mean feedback scores over all questions were significantly >0 for both the first survey (mean 1.16, SE 0.12; n=15 questions; t_21_=9.20; *P*<.001; 2-tailed *t* test) and the second survey (mean 1.29, SE 0.10; n=15 questions; t_17_=12.56; *P*<.001; 2-tailed *t* test). The scores remained largely consistent between the 2 surveys (Pearson *r*=0.87; *P*<.001; n=15). The strongest positive feedback was received for questions related to the ease of English used (mean 1.86, SE 0.10 in the first survey and mean 1.83, SE 0.12 in the second survey), the relatability of the examples (mean 1.23, SE 0.25 and mean 1.72, SE 0.13, respectively), the ease of using the CBT forms (mean 1.45, SE 0.18 and mean 1.22, SE 0.17), the engaging nature of the conversations (mean 1.36, SE 0.21 and mean 1.50, SE 0.20), the helpfulness of the *Learning* slides (mean 1.73, SE 0.10 and mean 1.67, SE 0.14), and the helpfulness of the *Learning* videos (mean 1.55, SE 0.13 and mean 1.67, SE 0.14). The features with the lowest ratings included the *PeerGroup*, which received weak positive feedback (mean 0.73, SE 0.23 and mean 0.61, SE 0.28) and the *buddy* feature, which received neutral feedback in both surveys (mean 0.09, SE 0.22 and mean 0.39, SE 0.20).

In the active control group, of the 7 participants who completed at least 3 modules, 7 (100%) and 5 (71%) participants submitted their first and second surveys, respectively. The survey questions were slightly different in the active control group (Table S5 in Multimedia Appendix 1); the first 6 questions judged their opinion on aspects they experienced directly, and the next 9 questions were asked in a prospective manner, for example, *I would prefer to have email reminders*. In the first 6 questions, the participants reported overall positive feedback (Figure 4B). In the 9 prospective questions, they showed interest in having only some of the proposed features, including conversations and game elements. Curiously, many features that the active control group participants thought they would not prefer—including SMS text messaging reminders, videos, and slides—were actually found to be useful by the experimental group participants who experienced the features (Figures 4A and 4B).

No participant reported any adverse events through the contact form on the website.

### Exploratory Analysis

To check if the content provided was engaging, we provided the experimental group participants with the option to provide feedback on the slides, videos, and *conversations* using *like* and *dislike* buttons. The slides, videos, and *conversations* were viewed 467, 205, and 1479 times, respectively, over all modules, of which nearly 17.1% (80/467), 19.5% (40/205), and 20.14% (298/1479) instances resulted in *likes* or *dislikes* feedback (Figures S4A, S4B, and S4C in Multimedia Appendix 1). We found that the feedback included more *likes* than *dislikes* for slides (mean 8.0 SE 2.30 likes vs mean 0, SE 0 dislikes; Wilcoxon W=45; n=10 slides; *P*<.001; Figure S4D in Multimedia Appendix 1); videos (mean 7.4, SE 1.51 likes vs mean 0.60, SE 0.54 dislikes; W=15; n=5 videos; *P*=.06; Figure S4E in Multimedia Appendix 1); and *conversations* (mean 1.88, SE 0.24 likes vs mean 0.12, SE 0.033 dislikes; W=6015; n=149 *conversations*; *P*<.001; Figure S4F in Multimedia Appendix 1). The participants also had the option of providing descriptive feedback on these elements. The subjective feedback was mostly positive, with participants frequently mentioning that they liked the given examples. One participant mentioned that they would have preferred to type their own answers in *conversations* (instead of choosing from prewritten text options). A word cloud created from the collated subjective feedback showed that the most frequently used words in feedback included *given*, *example*, *liked*, and *idea* (Figure S4G in Multimedia Appendix 1). TreadWill allowed participants to revisit previously completed *conversations* to refresh their memory; this option was used 17 times by the participants.

TreadWill allowed participants to attach one or more word tags from a list of 44 tags to posts in the *SupportGroup*. A word cloud of the tags used during the study revealed the topics that were most commonly discussed by the participants: *wasting time*, *loneliness*, *guilt*, *self-esteem*, and *trust* (Figure S5A in Multimedia Appendix 1). We also analyzed the entries made by the participants in the CBT forms (worksheets) to identify the common themes in their activities and concerns (Figures S5B and S5C in Multimedia Appendix 1). We checked the most commonly selected situations, thoughts, and beliefs from the lists presented to the experimental group participants. The most selected situation, thought, and belief were *I am concerned about my career,* *I should be doing something better*, and *If I don’t work very hard, I’ll fail*, respectively. (Figure S6 in Multimedia Appendix 1 presents the 10 most frequently selected situations, thoughts, and beliefs.)

All waitlist group participants had the option to use the experimental intervention once their participation in the waitlist group was complete. Of the 205 waitlist group participants, 70 (34.1%) signed up to use the experimental group (of which 64/70, 91.4% submitted the follow-up). Of these 70 participants, 7 (10%) completed at least 3 modules and 5 (7.14%) completed all 6 modules. These values were comparable with the completion rates in the experimental group participants. In addition, we calculated the reduction in PHQ-9 and GAD-7 scores from waitlist posttreatment time point to the primary end point for the participants who completed at least 3 modules. The reductions in PHQ-9 (mean 3.14, SE 1.14; n=7) and GAD-7 (mean 4.28, SE 0.75; n=7) scores were statistically similar to those of the experimental group participants.

## Discussion

### Principal Findings

We have presented the design of an unguided cCBT–based multicomponent intervention, TreadWill, aimed at high user engagement and universal accessibility. A fully remote RCT with 598 participants was performed to test the effectiveness of TreadWill in reducing depression- and anxiety-related symptoms. The results of the trial show that the full-featured (experimental) and the plain-text (active control) versions of TreadWill effectively reduced both PHQ-9 and GAD-7 scores for the participants who completed at least 3 modules compared with the waitlist control group. The number of participants who completed at least 3 modules in the experimental group was nearly 3 times more than in the active control group. The extra features included in the experimental version increased adherence compared with the active control version in terms of both the time of engagement and the number of modules completed. The results also showed that the number of modules completed correlated with the reduction in the symptom severity of a participant.

Two automated surveys presented during the intervention for taking participant feedback showed that the participants perceived TreadWill as useful and easy to use and found most of the interactive features helpful. In addition, the feedback provided by the participants using *like* and *dislike* buttons on different elements of the modules indicated that the participants found the content relatable and useful. Our target population was tech savvy and educated individuals (high school students, college students, and working young adults). We expected this target demographic to be comfortable with English to understand the material. We kept the language used in TreadWill simple enough for nonnative speakers to understand. The survey results confirmed that *Easy English* was one of the highest-rated features of TreadWill (Figure 4).

### Completer’s Analysis

An intention-to-treat analysis allows one to assess whether *assigning* a particular intervention helps the participant. In an intention-to-treat analysis, all participants assigned to the interventions are analyzed, regardless of their completion status; the missing data are imputed or carried forward from earlier observations. The missing data problem is manageable in most studies, in which participants are recruited and monitored by experimenters, and the participants generally have high intrinsic motivation or perceived psychological pressure (owing to the involvement of others) or receive compensation for participating in the study. However, in a web-based, remote intervention, the intention-to-treat analysis might not be suitable, as previously noted by Christensen et al [44]. The problem becomes even more severe when a study, such as ours, is completely unguided; there is no compensation for the participants, and there are no psychological barriers to joining or leaving the study at any time, just by using a smartphone. Although such open designs pose a problem for the intention-to-treat analysis, they are desirable in other aspects, as they mimic the real-life use patterns of smartphone-based self-help interventions.

An alternative analysis approach is to perform a completer’s analysis, in which the data of only those participants are analyzed who actually use the intervention. A completer’s analysis allows one to assess whether *completing* a particular intervention helps a participant. This is a more restricted claim compared with what can be made with an intention-to-treat analysis, especially from the perspective of a public health agency that has to decide which interventions to recommend to people. However, in emerging cases of smartphone-based self-help interventions for which an intention-to-treat analysis is not ideal, a completer’s analysis can be a reasonable alternative. This approach has also been used in previous studies either in isolation or in combination with an intention-to-treat analysis [30,44-46,64-68].

Another rationale for using an intention-to-treat analysis is that, in the presence of dropouts, including all participants in the analysis maintains the equivalence established among the different groups at the baseline. Although we performed a completer’s analysis, we found that the baseline equivalence was also maintained in our data. The baseline PHQ-9 scores for all the participants who were included in our primary analysis after removing dropouts remained similar (Kruskal-Wallis H(2)=1.11; *P*=.57; Figure 2A).

### Limitations

We did not require a clinical diagnosis of depression for including participants in the study because our goal was to create an accessible tool catering to both clinical and subclinical populations. Given that the prevalence of subclinical depression, defined as a score in the range of 5 to 9 on the PHQ-9, is fairly high at 15% to 20% [40,69,70], an unguided intervention can be immensely beneficial. We used only self-reported assessments for measuring symptom severity. Although self-reported assessments have their drawbacks [71], it was essential given the pragmatic nature of the study with an unguided intervention. For the same reason, we also included participants undergoing other treatments (42/598, 7% of our participants; Table 1). We used only 1 questionnaire each for assessing severity of depression and anxiety symptom. This decision was made keeping in mind that filling long questionnaires on the web is not a pleasant experience for users and might increase dropout rates [34]. In addition, while including multiple questionnaires for assessing the same disorder might improve validity, it also increases the risk of obtaining false-positive results by chance. Owing to the high dropout rate, we were unable to perform an intention-to-treat analysis. Although a completer’s analysis might be justified for a fully remote RCT, future work can evaluate TreadWill in a more traditional trial setting to assess intention-to-treat effects. Finally, our participants were young, mostly male, and tech-savvy college students, which reduces the generalizability of our results to the wider and much diverse population of India.

### Adherence Rates in cCBT Interventions

Deprexis, a well-evaluated intervention, reported a full adherence rate of 7.5% in a fully unguided evaluation [50]. The high adherence rates observed in trial settings often fail to translate into the real world [35]. In real-world studies, adherence can be very low: 5.6% in the study by Lara et al [72], 13.11% in the study by Morgan et al [73], and 5% in the study by March et al [74]. In a fully remote trial of an app-based intervention, Arean et al [47] reported that 57.9% of the participants did not even download their assigned apps. Similarly, in another study involving no human contact, Morriss et al [34] reported that only 57.3% of participants randomized to the experimental group signed up for the intervention and only 42.5% accessed it more than once. Morriss et al [34] further reported an attrition rate of 84.9% at the 3-week follow-up, with the attrition rate increasing at later follow-up points. In another recent study, Oehler et al [75] observed that the minimal dose was received by only 2.10% of the participants for the unguided version of iFightDepression. Guarino et al [76] also reported in a recent study that out of 2484 participants who signed up, only 562 started one of the modules, and the module completion rates ranged from 1% to 13% even by liberal definitions of module completion. The completion rates of web-based, self-help, and unguided cCBT interventions are comparable with the completion rates of massive open web-based courses, which have been reported to range from 3% to 6% [77,78]. The adherence rate observed for TreadWill, 12.1% (22/181) for moderate use and 5.5% (10/181) for full completion, is comparable with that reported in previous real-world studies. At this adherence level, TreadWill can benefit a significant number of people from the general population as a fully automated and scalable intervention. A web-based self-help intervention has a low opportunity cost; joining and dropping out of one intervention usually does not prevent a user from using another intervention; and trying out multiple apps before settling on one is a common behavioral pattern observed with smartphone apps. In addition, it is possible that the existing cCBT interventions and other digital mental health interventions do not provide help in the format that users expect on the web.

The 12.1% adherence rate for moderate use was observed in our study despite additional challenges compared with other studies. Every step in our study, from participant recruitment to assessment, was fully automated; the lack of human contact is known to affect the commitment of participants [34]. Christensen et al [64] evaluated the cCBT intervention MoodGYM in 2 different settings: in a *trial setting* in which participants were called every week by human guides and provided instructions on completing the intervention, the completion rate for all 5 modules was 22.5%, but in an *open setting* (with no human contact), only 0.49% (97/19,607) of the participants completed the intervention. The intervention used in both cases was identical; the only difference was the interaction between participants and experimenters in the *trial setting*. This study shows that although it is possible to obtain higher completion rates in standard trial settings, these rates do not translate into real-world settings. Therefore, we used a pragmatic trial with no human contact, and even though the adherence rates are low, they are expected to be a more faithful representation of the real-world completion rates. Furthermore, it has been reported that male sex and young age significantly increase the chance of dropout [79]. The average age of our participant group was 23.6 (SE 0.17) years, and 79.9% (478/598) were male, which could have contributed to the dropout rate. Contrary to the practice of giving money or gift cards to participants [47,80-82], we did not reward participants for submitting assessments or for participation. In several studies [47,80-82], participants were paid even after submitting the baseline assessments. The practice of paying participants is likely to influence adherence to the intervention owing to the rule of reciprocity [83] and influence the assessment responses. Participants getting paid might feel that they owe it to the researchers to use the program and try to give answers in the assessments that they think the researchers expect. Not giving a reward also supports our pragmatic trial design; as in the real world, paying participants to use the intervention will be unsustainable.

The generally positive comments that we received from the participants on the content (Figure S4 in Multimedia appendix 1) and various features of the intervention in the 2 surveys (Figure 4) suggested that the user dropout was not because of the unacceptability of the intervention. To check this further, we compared the survey responses of the experimental group participants who had completed all 6 modules with those who dropped out before completing 3 modules but completed a survey. The average feedback score was not lower in the *dropout* group than that in the *completer* group (Figure S7 in Multimedia Appendix 1).

### Implications

Our study shows that even in low-resource settings, a cCBT-based intervention without expert support can help users who at least partially complete the intervention. This implication is immensely encouraging, as the number of mental health professionals is extremely low in India [84,85]. The reduction in PHQ-9 scores in our study was 2.73 for users who completed at least 3 modules and 4.20 for users who completed all modules. This level of reduction in a low-threshold intervention, with the potential to have a population-level impact, can be considered clinically significant [32]. Our study established TreadWill as a potential population-level intervention. This is among the largest studies conducted in India on digital mental health [86-89]. Our study is also the first fully web-based trial conducted in India and provides a template for conducting web-based trials for other mental health conditions in the country. Future work should focus on strategies, such as using gamification, serious games, or chatbots to build therapeutic alliance, to improve adherence to self-help interventions. Future work can also focus on making the intervention more similar to general web-based apps, so that users receive help in the formats with which they are familiar. In this study, we created tailored content for high school and college students and working professionals. Future work should also target the unemployed population and other susceptible groups.

## Appendix

Multimedia Appendix 1 Supplementary figures and tables.

Multimedia Appendix 2 CONSORT-EHEALTH (V 1.6.1) Checklist.

## Abbreviations

CBT: cognitive behavioral therapy
cCBT: computerized cognitive behavioral therapy
GAD-7: Generalized Anxiety Disorder-7
LMICs: low- and middle-income countries
PHQ-9: Patient Health Questionnaire-9
RCT: randomized controlled trial

## Data Availability

The deidentified data analyzed in this study are available from the corresponding author upon reasonable request.
